# Offensive Breath

**Published:** 1873-03

**Authors:** D. C. Hawxhurst


### Offensive Breath.

BY. D. C. HAWXHURST.

I walked one summer's evening along a green lane strewn with apple blossoms. Approaching the door of my home, a low hum of voices fell upon my ear; at the threshold I detectəd a subtle fetor in the air. Although it was deep twilight I knew whom to salute when this odor presented itself.

A young lady acquaintance, marked by an unbearable breath, and distinguished for her beauty, was making an evening call. A breath more execrable than hers I have seldom known to come from so beautiful a face, and a beautiful face more completely bereft of the legitimate attractions of beauty, through the mere presence of something disgusting, I have seldom encountered in my practice.

This young lady ultimately became my patient. It was my experience with her that spurred me up, more than any other single thing, to a study of the causes, and the conditions of fetid breath.

It is folly for the practitioner to underrate the influence which a bad breath may exert in driving his patients from him. Under my own observation several operators have suffered seriously from this cause.

The first feeling that comes into the mind on smelling a disagreeable odor is a desire to get away from it. The dentist whose breath is infected with odors that disgust, must not be surprised if his patients adopt speedy means of putting themselves at a safe distance from this infection. No other single influence can equal it in repelling those on whom he is to rely for his support.

I am acquainted with an intelligent and courteous operator who carries an offensive breath, and who has seen his patients drop away from him one by one, until at last he cultivates the mechanical part of dentistry almost exclusively; nor do I know whether he is to this day aware how slight a cause has thrown him into the practice of that specialty.

The olfactory nerve becomes dulled to the impression of accustomed odors. Dentists do not perceive their own offensive emanations any more than other men. Shoemakers do not realize the odor of leather about their shops; grooms do not know how their clothes smell; the customer, and not the proprietor, is most alive to the odors about an apothecary shop; and the tobacco eater, enjoying his quid or pipe, is the only one who does not know what a sickening fetor these articles add to his breath. The truth is no one detects the smell he is used to, and by consequence, since each one is accustomed to his own breath, he will regard it as odorless, though its vapors be loaded with a dozen different stenches.

The dentist should never operate unless he knows that his breath is pure. He should keep his spittoon sweet; he should keep his rooms well supplied with oxygen; he should by changes of linen and bathing render his person offenseless; but above all he should exercise a constant watchfulness over his breath.

To pack gold for an hour and a half, and during the whole time breath a stream of fetid odor directly into the face of the patient, is a gross insult. If an apology could ever make a bad matter better it should be made here. An apology is a poor indemnity, however, for the nervous prostration, physical exhaustion, and nausea, that often result from breathing a foul breath.

Uncleaned finger nails and grimy hands will not cause disgust so strong, nor give offense so deep, as the breathing of disagreeable odors. If neglected hands offend the sight, the patient may withhold his glances; against a bad breath he can make no defense during protracted operations, and the dose becomes doubly disgusting from excess.

To diagnose his own breath should be one of the operators daily duties. It will not do for him to assume that it is sweet to-day, because it was sweet yesterday. The matter of the animal organism undergoes ceaseless change, and the exhalations vary continually. The breath does not therefore preserve an unchangeable composition. During great vicissitudes of emotional and nutritive states, it may present different odors, sweet or foul, at each half hour of the day; especially after a meal of food that is highly carbonaceous, or rich in the essential oils, is there likely to be a change in the breath.

By the time an operator has taken his breakfast, enjoyed a chat with his wife and children, and made his way to the office, the first absorbed products of digestion will have become a part of the blood. Though his wife may have assured him that his breath is sweet, there has been ample time for some article of food, as for instance a little cellery, especially if his digestion be imperfect, to communicate its odor to the breath. Or perhaps a cigar, taken on the way, may have done the mischief. I have even known the mouth to be rendered foul by the decomposition of a trace of butter, lingering about and between the teeth, introduced there while ordering the day's groceries. It may at any rate be set down as certain, that frequent tests of the breath are necessary. Self-examination will not prove efficient. Let the dentist who would not be disagreeable to his patient, avail himself of the kindly offices of some trustworthy friend.

If an examination reveals a state of the breath, which requires attention, let the practitioner not approach his patient until all sources of offence are removed. That should be an urgent case which could induce him to break this rule. The man who would wish to be loved, as well as respected by his patients, and indeed the man who desires to have and to hold the more refined and better class of patients, will seldom depart from it.

When the practitioner has acertained that his breath is in a state that must unfit him to appear before his patient, at least for the present, it becomes his first duty to trace the vitiating odor to its cause. Whatever vile thing it may be, he will make little progress in removing it, until he discovers its source. This will be found in the mouth, nose, throat, or chest.

Neglected teeth are nearly always offensive. Various alimentary substances find a lodgment in the interstices about the mouth and teeth, and unless removed in time to prevent fermentative changes, are certain to present putrefactive compounds to the currents of air as they pass and repass in talking.

The obvious significance of this is that the operator should often look a little after the condition of his mouth; he should daily cleanse and polish the labial, lingual, approximal, and grinding surfaces of every one of his teeth; if necessary he should brush off deposits upon the mucous membrane. There should be no carious teeth, no neglected roots, no calcarious deposits in the mouth of the dental practitioner.

At intervals we find men in our profession who chew or smoke tobacco. I am convinced that the "coming dentist" will not chew tobacco any more than he will chew assafœtida. The odor arising from this drug, especially from a pipe or cigar, is penetrating, and often clings obstinately to the teeth and oral surfaces after a second or third brushing; and there is a sickening fetor about it after an hour or more has elapsed, which is not observed immediately after indulging in it. The mustache, beard, hair, skin and clothing, condense it on their surfaces, and confine it in thelr meshes.

The warm air of a room, or the heat from a stove, may volatilize, and render sensible, remaining traces of this odor, even after what was regarded as a careful cleansing. The practitioner who has smoked a cigar can not be put through the requisite processes of purification, bathing, fumigation, and disinfection, in any such manner as will make it permissible for him to enter the presence of a patient of refined sensibilities, short of several hours of conscientious and pains-taking labor.

The breath of a chewer has peculiarities of its own; it is rank and strong; it is different from every other smell; it hold its fetor long after the quid has been cast away, and the mouth thoroughly rinsed. I have seen a practitioner apologize for his quid, throw it out, rinse his mouth, and resume his work. It is to be hoped that no dentist will hereafter make so forlorn an effort to be agreeable to his patient. It is doubtful whether the offensiveness of the breath was very much modified by the removal of the tobacco.

The mucous membranes, glands, and parts about the mouth and throat, absorb the drug, and give off its odors, while the blood itself will for a time continue to throw out through the lungs portions that have been introduced into the circulation by capillary absorption.

Tobacco is neither food nor medicine, and may be entirely abstained from without detriment to the health; nor can a professional man be regarded as out of fashion because he does not chew or smoke. The purest people in the best circles of society do not use tobacco.

In the fauces there sometimes occur little nodular bodies, made up of cheese-like matter, which constitutes the source of a peculiar fetor. A nasal douche syringe may aid in their removal. Snuffing water through the nose will often dislodge them. In some cases it will be necessary to gargle the throat, and throw water upward into the nose, or snuff it backward through the nostrils and over the schneiderian membrane.

If there be cartarrh, some disinfectant may be employed with the syringe to apply it. All those parts over which the currents of air sweep, on their way to and from the lungs, should be rendered clean and odorless. Before the practitioner may approach his patient he must assure himself of this. After all else has been done that can be, a few minutes breathing of pure cold air will complete the purification. The atmosphere is one of nature's best deodorizers. Appropriate and discriminating attentions of this kind will often conquer a most obstinate breath.

The practitioner who would rid himself of an offensive breath, will first examine and purify all those parts that lie above the larynx. Four-fiths of all cases will thus be cured, or very much aleviated. Their management is not generally difficult. A frequent repetition of the cleansing process is often necessary, especially if the membranes of the throat or nose, the bones of the face, or the maxillary sinuses, are the seat of chronic disease.

The damaging effect which the last mentioned affections occasionally exert on the breath is immense. Such diseases, unless susceptible of complete and permanent cure, can not but be regarded as serious impediments to the practice of dentistry, since it is rare in the extreme that any one who suffers from them can be induced to exercise the care and attention requisite to preserve the breath sweet.

Bad breath from the above mentioned sources is of comparatively easy diagnosis. None of the parts are deep-seated, nor very difficult of examination. Cases will often occur, however, in which all parts above the larynx seem to be in health, and the source of the offending odor can be traced to the lungs themselves. To fix with certainty upon the exact cause, which is operative in such cases, will often require the skillful diagnostitian to summon all his resources; and in their cure he will meet with peculiar and embarrassing difficulties.

In cases of offensive breath originating in the lungs, we have not in general a local source of foul odor to which we may apply a purifying agent. The impurity exists in the blood, and permeates every tissue of the organism; it communicates an odor to the skin; it impregnates about twenty-five pounds of circulating fluid. An ounce of blood drawn from the veins will, in such a case, offend like the breath itself. In thus smelling the odor in the blood, instead of in the breath, we are only taking it directly, and at first hand. It is but the work of a moment for it to escape from the blood into the air passages, and manifests itself as a morbid agent in an offensive breath.

If the practitioner of dentistry shall discover that his breath is foul from an impure blood, he must no longer work at the chair until he is recovered, he must, himself, for a time, play the role of patient.

The treatment may include all the legitimate means of blood-purification. The functions of excretion should be encouraged up to the point of free activity; it is more than likely that one or more of them are sluggish, and do not withdraw the requisite amount of waste and foul matter from the system.

The diet too should receive attention. Pure foods in moderate quantity furnish very little material out of which a bad breath can be made. Known polluters of the breath, such as beer, wine, sour-krout, and hard cider, may easily be entirely avoided.

But the question of a bad breath orginating in the lungs, demands a separate treatment. In this essay I have intended to give one or two hints towards its solution, reserving its fuller treatment for a subsequent paper.

